# A Piece of the Neuroeconomic Puzzle: Effort-Based Decision-Making and Physical Activity—A Systematic Review

**DOI:** 10.3390/bs16091508

**Published:** 2026-08-27

**Authors:** Jovana Vitošević, Milica Filipović, Damljan Bogićević, Miloš Popović, Biljana Vitošević, Igor Ilić, Katarzyna Sterkowicz-Przybycień, Slobodanka Dobrijević, Tijana Purenović-Ivanović

**Affiliations:** 1 Department of Business Studies, Toplica Academy of Applied Studies (TASS), 18420 Blace, Serbia; jovana.vitosevic@vpskp.edu.rs; 2 Faculty of Sport and Physical Education, University of Pristina in Kosovska Mitrovica, Dositeja Obradovica bb, 38218 Leposavic, Serbia; milos.popovic@pr.ac.rs (M.P.); biljana.vitosevic@pr.ac.rs (B.V.); igor.ilic@pr.ac.rs (I.I.); 3 Faculty of Medical Sciences, University of Kragujevac, Svetozara Markovica 69, 34000 Kragujevac, Serbia; damljanbogicevic@yahoo.com; 4 Department of Gymnastics and Dance, Institute of Sport Sciences, University of Physical Culture in Krakow, 31-571 Krakow, Poland; katarzyna.sterkowicz@awf.krakow.pl; 5 Faculty of Sport and Physical Education, University of Belgrade, Blagoja Parovica 156, 11000 Belgrade, Serbia; slobodanka.dobrijevic@fsfv.bg.ac.rs; 6 Faculty of Sport and Physical Education, University of Nis, Carnojevica 10a, 18000 Nis, Serbia; tijanapurenovic@gmail.com

**Keywords:** effort-based decision-making, effort discounting, physical activity, exercise behavior, neuroeconomics, subjective value, motivation

## Abstract

Regular physical activity protects body and mind, yet most adults remain insufficiently active—a shortfall increasingly understood not as a failure of knowledge but as a problem of decision, in which an immediate cost in physical effort is weighed against delayed, uncertain rewards. Neuroeconomics frames this valuation, yet the effort cost most intrinsic to exercise has drawn little attention beside delay. This systematic review synthesised evidence on the relationship between effort-based decision-making and real-world physical activity in both directions. Following PRISMA 2020 and a pre-registered protocol, Scopus, Web of Science, and PubMed/MEDLINE were searched up to June 2026; records were screened against two criteria—an effort-based decision construct and an exercise outcome—independently and in duplicate, and appraised with the MMAT. Of 485 database records, nine studies met eligibility; backward citation searching added three (twelve in total). Evidence emerged for associations in both hypothesised directions: steeper effort discounting accompanied lower activity, while exercise appeared to reshape effort valuation. The largely observational designs, however, preclude causal inference. Most used real, enacted exercise; one recent study added hypothetical choices. Effort-based decision-making emerges as a neglected yet malleable piece of the neuroeconomic puzzle of activity, with clinical and methodological implications.

## 1. Introduction

Regular physical activity is among the most effective protective behaviours for physical and mental health, with benefits spanning cardiovascular, metabolic, mood, and cognitive function. And yet, despite a public-health message repeated for decades, a substantial share of adults remain insufficiently active, with roughly one in three falling short of recommended levels worldwide (Guthold et al., 2018). The puzzle, then, is not truly one of knowledge—most people know that movement is good for them—but one of decision. Day after day, the choice to be active asks something deceptively difficult: to pay an immediate and certain cost in physical effort now, in return for rewards that are delayed, diffuse, and uncertain. Understood in this light, the persistence of inactivity appears less as a failure of information than as a recurring problem of valuation; to make sense of it, we must turn to the science of how the brain weighs what is worth doing, a view toward which recent syntheses of the psychophysiological foundations of physical activity increasingly converge (Gerber et al., 2025).

Neuroeconomics is the discipline tailored to precisely this question. Sitting at the meeting point of economics, psychology, and neuroscience, it describes how the brain assigns a subjective value to each available option by weighing its anticipated rewards against its anticipated costs (Bossaerts & Murawski, 2015; Krajbich & Dean, 2015; DeStasio et al., 2019). Within this framework, research on intertemporal and effort-based choice has repeatedly identified two costs that reduce the subjective value of a reward in discounting paradigms: the delay before it arrives, and the effort required to secure it. Klein-Flügge et al. (2015) showed that these two costs are not interchangeable but discount reward through dissociable computational processes, a finding that grants effort the status of a distinct cost in effort-based choice.

Of these costs, physical effort is the one most intrinsic to exercise, for it is paid in the body and in the present moment. Over the past two decades, dedicated neuroscience reviews have characterised a network implicated in effort-based valuation—encompassing dopaminergic signalling together with the anterior cingulate cortex, the ventral striatum, and the ventromedial prefrontal cortex—that is thought to integrate the cost of effort into an overall sense of what an action is worth (Rigoli & Pezzulo, 2022; Bogdanov & Pizzagalli, 2026). We draw on this literature as a background; the neural mechanisms of effort valuation were not themselves the object of the present systematic search. This valuation is dynamic, shifting with fatigue, reward, and individual disposition, and it is increasingly recognised as a core determinant of whether effortful action is initiated and, more tellingly, sustained (Audiffren et al., 2022). It is this automatic gravitation toward the least effortful option that the Theory of Effort Minimization in Physical Activity (TEMPA; Cheval & Boisgontier, 2021) places at the very heart of our struggle to be active.

For all its sophistication, however, this science has been built almost entirely upon abstract laboratory proxies—the squeeze of a handgrip, the press of a button, the choosing between hypothetical options—designed for experimental control rather than ecological fidelity (Cheval et al., 2025; Stähler et al., 2025). Running in parallel, and rarely in conversation with it, the exercise-adherence literature has explained physical activity largely through attitudes, intentions, and self-efficacy, frameworks that seldom incorporate the formal cost–benefit computation lying at the heart of neuroeconomics (André et al., 2024). Where a bridge between the two has been attempted, it has rested mostly on delay discounting, with several studies linking steeper temporal discounting to lower activity (Tate et al., 2015; Sofis et al., 2017; Berardi et al., 2024). The effort construct, the very cost most intrinsic to the act of exercising, has remained comparatively unexamined in relation to real-world behaviour. Whether the effort valuation so carefully characterised in the laboratory genuinely translates to the activity people perform in their daily lives is, at present, an open question, and one that no systematic review has yet addressed.

The importance of this question is only amplified in clinical contexts, where diminished willingness to invest effort and reduced engagement in physical activity so often travel together. Altered effort-based decision-making has now been documented across a range of conditions: depression (Hird et al., 2024), schizophrenia (Décombe et al., 2020), Parkinson’s disease (Colón-Semenza et al., 2021), anorexia nervosa (King et al., 2025), attention-deficit/hyperactivity disorder (Bernacer et al., 2025), and the post-stroke state (Erfanian Abdoust et al., 2024), many of which are themselves marked by entrenched inactivity. A clearer understanding of how effort is valued, and of how that valuation is coupled to movement, may therefore help to explain why physical activity is so difficult to initiate in these populations, and how supportive interventions might be more thoughtfully tailored to them.

The present review draws these threads together. Its aim is to systematically synthesize the evidence on the relationship between effort-based decision-making and real-world physical activity or exercise behaviour, examined in both directions: whether the way individuals value effort predicts how active they are, and whether exercise, in turn, reshapes the valuation of effort itself. Recognising that this young field spans both genuinely enacted activity and more simulated or hypothetical decisions, we have deliberately designed the synthesis around two tiers, studies in which exercise is real and performed, and those in which it is simulated or imagined so that the ecological reach of the evidence may itself be brought into view. Accordingly, the review pursues three objectives: (1) to synthesise the evidence linking effort-based decision-making to physical activity and exercise behaviour; (2) to examine whether the neural correlates of effort valuation are related to activity behaviour; and (3) to map the methodological architecture of this literature, its populations, tasks, and measures, and to appraise the ecological validity of the laboratory paradigms on which it rests. In doing so, the review follows the PRISMA 2020 framework and a pre-registered protocol and offers a systematic account of effort-based decision-making as a missing piece in the neuroeconomic understanding of why we move, or fail to move.

## 2. Materials and Methods

### 2.1. Protocol, Registration, and Reporting

This systematic review was conducted and is reported in accordance with the Preferred Reporting Items for Systematic Reviews and Meta-Analyses (PRISMA) 2020 statement (Page et al., 2021). The review protocol was registered with the International Platform of Registered Systematic Review and Meta-Analysis Protocols (INPLASY; registration number INPLASY202660143, available at https://doi.org/10.37766/inplasy2026.6.0143). The review adheres to that pre-specified design. The single departure from the registered protocol is that the Tier 1 category was subdivided post hoc into Tiers 1a–1c (distinguishing habitual activity, athletic training status, and enacted laboratory or acute exercise) to aid the narrative synthesis; all other elements, including the pre-specified Mixed Methods Appraisal Tool (MMAT, Hong et al., 2018) and the two-tier framework, were applied as registered. The completed PRISMA 2020 checklist, the full search strategies (Supplementary Table S1), the citation-search tracker (Supplementary Table S2), and the data-extraction table (Supplementary Table S3) are provided as Supplementary Materials.

### 2.2. Eligibility Criteria

Eligibility was framed according to a PECO structure. The population comprised human participants of any age and health status, spanning both healthy and clinical samples. The exposure of interest was effort-based decision-making, operationalised as effort discounting, effort-cost sensitivity, the willingness to expend effort for reward, or the neural valuation of physical effort, assessed through behavioural tasks and/or neural and physiological indices. These operationalisations constitute a related family of effort-valuation constructs rather than a single, interchangeable measure; they differ in method (behavioural choice or discounting tasks, momentary benefit–cost ratings, self-report valuation scales, and neural indices), and we did not assume psychometric equivalence across them. Accordingly, studies are grouped in the synthesis by construct and measurement approach, and this heterogeneity is treated as a limitation (Section 4.6). The outcome was real-world or habitual physical activity and exercise behaviour, captured through device-based measurement, validated self-report, athletic training status, or exercise performed as the effortful action itself. A PECO (rather than PICOS) structure was adopted because the review concerns an exposure—effort-based decision-making—and its association with physical activity, not a healthcare intervention; PICOS, which centres on interventions, was therefore not appropriate, and the PEO structure was extended to PECO because several eligible designs incorporate an explicit comparison. The comparator was defined by design: for group and case–control comparisons, the contrasting group (e.g., athletes vs. non-athletes; patients vs. controls) or, for pre–post designs, the pre-exercise state; for experimental studies, the control condition or lower-effort option (e.g., low- vs. high-cognitive-load; exercise vs. sedentary session; low- vs. high-effort choice); and for cross-sectional and scale-based studies without a discrete comparison group, variation across the exposure itself (e.g., steeper vs. shallower discounting, or higher vs. lower effort-valuation scores, between- or within-person). Studies without a separate control arm thus satisfy PECO through contrast along the exposure continuum, consistent with the use of PECO in syntheses of observational evidence.

Two conditions had to be satisfied jointly for a study to qualify. First, the study had to measure a genuine effort-based decision-making construct; second, exercise or physical activity had to be measured or manipulated, with exercise serving as the explicit object of the decision rather than as incidental framing. Studies were eligible in either direction of the relationship: those examining whether effort valuation predicts activity, and those examining whether exercise reshapes effort valuation. To capture the ecological breadth of this young field, eligible studies were further classified into two tiers, with Tier 1 subdivided to reflect its differing ecological character. Tier 1 comprised enacted exercise, distinguished into the following: (1a) habitual, real-world physical activity measured by device or validated self-report; (1b) athletic training status; and (1c) enacted laboratory or acute exercise (single bouts, cycling tasks, or short interventions). Tier 2 comprised simulated or hypothetical exercise decisions. We emphasise that enacted exercise is not automatically more ecologically valid than a simulated decision: ecological validity is multidimensional (naturalistic context, time frame, behavioural consequence, and measurement objectivity), and a controlled laboratory exercise task (Tier 1c) may resemble everyday activity less than a hypothetical choice sampled in a naturalistic setting. The tiers therefore index the enacted–simulated dimension specifically, rather than overall ecological validity. Although real-world, enacted physical activity was our primary focus, eligible studies were deliberately allowed to span both of these tiers, in order to capture the full range of effort-based decision-making paradigms applied to exercise.

Empirical, peer-reviewed original research articles using observational (cross-sectional or cohort) or experimental and interventional designs were eligible. We excluded studies lacking an effort-based decision-making construct (for example, investigations of the effects of exercise on cognition with no valuation component), studies relying on abstract laboratory effort proxies with no exercise or physical-activity outcome, animal studies, and non-empirical sources (reviews, theoretical and opinion articles, editorials, commentaries, conference abstracts, and book chapters), which were retained only for contextual background and for reference-list screening. Only English-language records were considered and no date restriction was imposed.

### 2.3. Information Sources

Three multidisciplinary bibliographic databases were searched, selected to span the neuroscientific, psychological, and sport-science literature in which this work is dispersed: Scopus, the Web of Science Core Collection, and PubMed/MEDLINE. All searches were executed until June 2026, with no lower date limit (from each database’s inception to the search date). Searches were run on the following platforms: Scopus (Elsevier, Amsterdam, The Netherlands), the Web of Science Core Collection (Clarivate, London, UK), and PubMed (U.S. National Library of Medicine, Bethesda, MD, USA). APA PsycINFO and SPORTDiscus were not available through our institutional access. The complete search strategies—platforms, search fields, Boolean strings, filters, exact dates, and the number of records retrieved from each database—are reported in Supplementary Table S1. Backward and forward citation searching was conducted in June 2026 and identified three additional eligible studies. The database searches were not updated between the original search and manuscript submission; given the recency of the search and the currency added by forward citation searching, no update was undertaken.

### 2.4. Search Strategy

The search strategy was built upon two concept blocks combined with the Boolean operator AND, with terms within each block joined by OR and truncation applied to capture word variants. The first block represented effort-based decision-making (e.g., “effort-based decision*”, “effort discount*”, “effort cost*”, “effort expenditure”, “effort allocation”, “neuroeconomic*”); the second represented exercise and physical activity (e.g., exercise, “physical activity”, “physical exercise”, “exercise training”, “physical fitness”, sport*, walking, running, “sedentary behave*”). Delay and temporal discounting were deliberately excluded as search constructs and treated as background, so as to keep the review centred on the effort cost most intrinsic to exercise. A neural-mechanism block was intentionally not combined into the strategy, since doing so would have removed the behavioural studies central to the question. Searches were limited to original research articles and reviews and to English-language records; no filter for full-text availability or open access was applied, so as not to bias retrieval by accessibility.

### 2.5. Selection Process

**Figure 1:**
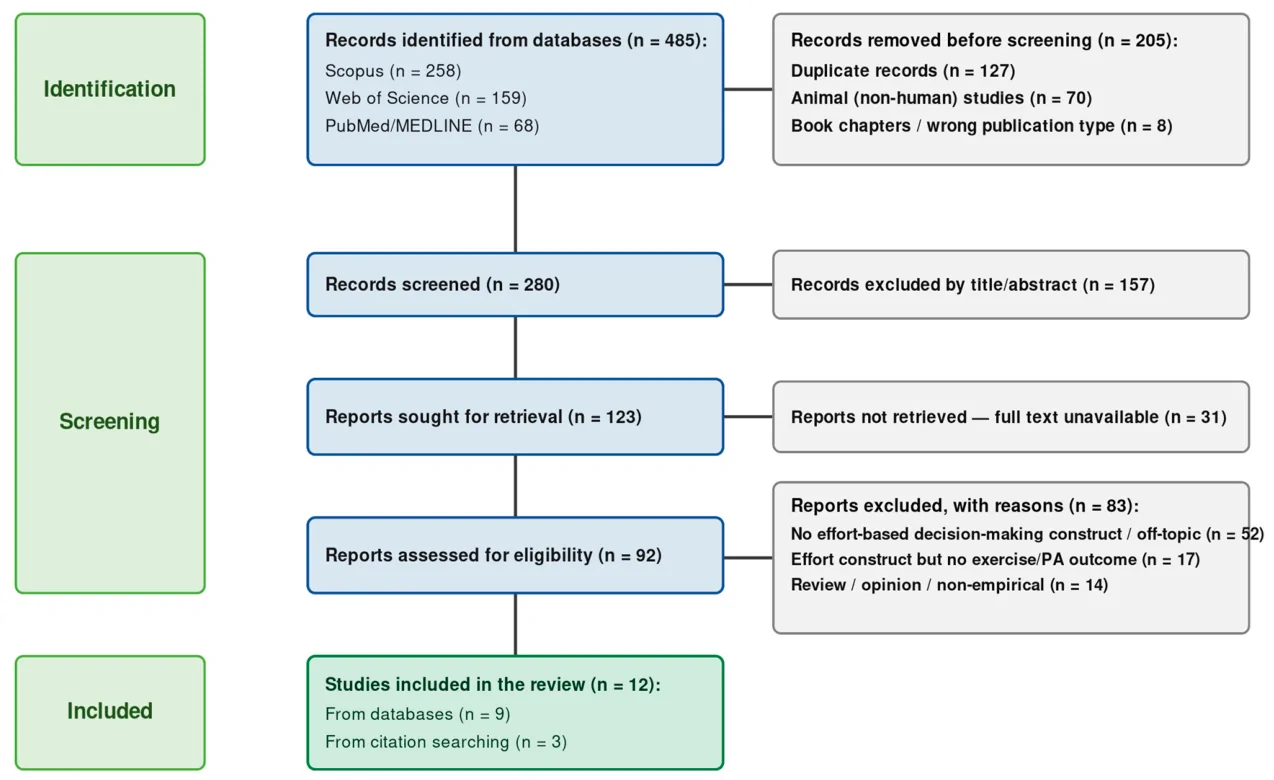
PRISMA 2020 flow diagram of the identification, screening, and inclusion of studies. Records identified through database searching (Scopus, Web of Science Core Collection, and PubMed/MEDLINE) and through backward and forward citation searching are shown, together with the reasons for exclusion at each stage.

All retrieved records were imported into a reference manager and de-duplicated. Before screening, ineligible publication types (book chapters, editorials, and similar) were removed using database document-type fields with manual verification, and animal (non-human) records were identified from the title and abstract—for example, studies explicitly conducted in rats or other animals—supplemented by the PubMed animal filter where available, and were removed before eligibility screening. Titles and abstracts of the remaining records were screened against the eligibility criteria, after which the full texts of potentially eligible records were sought through the institution’s subscription access and holdings; a total of 31 of the 123 reports sought could not be obtained—their full texts lay behind subscription barriers not covered by our institutional access, despite our attempts to retrieve them—and were recorded as not retrieved (Figure 1). The review was conducted by three groups of reviewers, each working in duplicate, so that every record and study was assessed independently by two reviewers. Title and abstract screening, full-text eligibility assessment, data extraction, and methodological (MMAT) appraisal were each performed in this way. Prior to independent assessment, the reviewers calibrated their application of the eligibility criteria on a common subset of records. Any disagreements—within or between groups, at any stage—were resolved by the two most experienced reviewers (B.V. and M.F.). A formal inter-rater agreement statistic (e.g., Cohen’s κ) was not computed; agreement was reached through discussion and adjudication by the two senior reviewers. Records excluded at the full-text stage were recorded together with their primary reason for exclusion. The complete selection process, including the number of records identified, screened, retrieved, and assessed, is summarised in the PRISMA 2020 flow diagram (Figure 1).

### 2.6. Data Extraction

Data were extracted using a piloted, standardised form. For each included study, we recorded the following: bibliographic details and country; study design; sample size and participant characteristics (age, sex, and health or clinical status); the effort-based decision-making task and its parameters; any neural or physiological measure; the physical-activity or exercise measure and its metric (device-based or self-reported); the direction of the relationship examined; the assigned tier; and the principal findings and effect estimates. Data extraction was carried out in duplicate, following the reviewer procedure described in Section 2.5.

### 2.7. Risk of Bias and Quality Appraisal

The methodological quality of the included studies was appraised using the Mixed Methods Appraisal Tool (MMAT), version 2018 (Hong et al., 2018). MMAT was selected because the included studies span randomised, non-randomised, quantitative descriptive, and mixed-methods designs, all of which are accommodated within a single, design-sensitive instrument. Following two screening questions common to all designs, each study was rated against the five criteria specific to its design category, with every item judged as ‘yes’, ‘no’, or ‘cannot tell’. In keeping with the developers’ guidance, the ratings were not collapsed into a single summary score; instead, the rating for each individual criterion is reported (Table 1). Each study was appraised in duplicate, following the reviewer procedure described in Section 2.5. Study quality was used to inform the synthesis and interpretation of the findings but was not applied as a threshold for exclusion.

### 2.8. Data Synthesis

Given the anticipated heterogeneity across populations, effort paradigms, and physical-activity measures, a structured narrative synthesis was undertaken in preference to meta-analysis. Findings were organised along two intersecting dimensions: by the two ecological tiers (real-world and enacted versus simulated or hypothetical exercise) and by the direction of the relationship (effort valuation predicting activity versus exercise reshaping effort valuation). Within these, studies were further grouped by the type of effort measure—behavioural decision or discounting tasks, momentary benefit–cost ratings, self-report effort-valuation scales, and neural or physiological indices—and by the nature of the activity measure. The ecological validity of the effort paradigms, how closely each resembled genuine exercise, was carried through the synthesis as a cross-cutting theme.

## 3. Results

### 3.1. Study Selection

The database searches, executed until June 2026 across Scopus, the Web of Science Core Collection, and PubMed/MEDLINE, returned 485 records (Scopus, *n* = 258; Web of Science, *n* = 159; PubMed/MEDLINE, *n* = 68). Before screening, 205 records were removed—127 duplicates, 8 book chapters or other ineligible publication types, and 70 animal studies—leaving 280 records for title and abstract screening. Of these, 157 were excluded as clearly irrelevant, and 123 reports were sought for retrieval. Thirty-one could not be obtained—their full texts lay behind subscription barriers not covered by our institutional access, despite our attempts to retrieve them—and the remaining 92 were assessed in full text. At this stage, 83 reports were excluded with reasons—most commonly the absence of a genuine effort-based decision-making construct or of an exercise or physical-activity outcome, together with non-empirical sources retained only as background—leaving nine studies that satisfied all eligibility criteria via the database search. Backward and forward citation searching of the included studies and of the key reviews then identified three further eligible studies (Bieleke et al., 2025; Cheval et al., 2024; Taylor et al., 2026), bringing the total to twelve included studies; additional candidates (e.g., Brown & Bray, 2019) were assessed and retained only as a background. Several apparently relevant reports were excluded for lacking an effort-based decision-making construct (Greenhouse-Tucknott et al., 2025; Filipas et al., 2021; Jonker et al., 2010; Holgado et al., 2025; Bray & Harris, 2025; Morris et al., 2022; Wellings et al., 2025). The complete selection process is depicted in the PRISMA 2020 flow diagram (Figure 1).

### 3.2. Characteristics of Included Studies

The characteristics of the twelve included studies are summarised in Table 1. They were published between 2018 and 2026 and were conducted in Spain, Germany, the United Kingdom, Canada, France, Switzerland and the United States. The designs were notably heterogeneous, encompassing a randomised laboratory experiment (Harris & Bray, 2019), a within-subject randomised crossover (Wardle et al., 2018), a longitudinal pre–post study (Bernacer et al., 2019), cross-sectional and case–control comparisons (Chong et al., 2018; Bernacer et al., 2025; Colón-Semenza et al., 2021), an intensive observational study pairing ecological momentary assessment with accelerometry (Harris & Bray, 2023), a mixed-methods experiment (Harris & Bray, 2021), an electrophysiological feasibility study (Renoud-Grappin et al., 2025), a large-scale scale-development and validation study (Bieleke et al., 2025), a multi-study validation of the Physical Effort Scale (Cheval et al., 2024), and a three-study forced-choice experiment on effort-based exercise choices (Taylor et al., 2026). Sample sizes ranged from 19 to 1267 participants. Both healthy and clinical populations were represented: most studies recruited healthy adults or athletes, while two examined clinical groups, adults with attention-deficit/hyperactivity disorder symptoms (Bernacer et al., 2025) and people with Parkinson’s disease (Colón-Semenza et al., 2021). Effort was most often operationalised through the Effort-Expenditure for Rewards Task or related effort-discounting paradigms, while physical activity was captured through device-based measurement, validated self-report, athletic training status, or exercise performed as the effortful act itself. Notably, eleven of the twelve studies fell within Tier 1 (real-world or enacted exercise); the exception, Taylor et al. (2026), additionally employed hypothetical exercise choices, furnishing the first Tier-2 evidence. Within Tier 1, the studies were heterogeneous in ecological character, ranging from habitual, device- or self-report-measured activity (Tier 1a; e.g., Harris & Bray, 2023; Bernacer et al., 2025), through athletic training status (Tier 1b; Chong et al., 2018), to enacted laboratory or acute exercise (Tier 1c; e.g., Wardle et al., 2018; Renoud-Grappin et al., 2025; Colón-Semenza et al., 2021); these differ in naturalistic context, time frame, and measurement objectivity, and the tier labels index the enacted–simulated distinction rather than ecological validity as such. Numerical effect estimates and their precision for each study, together with participant characteristics, adjustment variables, and study-level limitations, are reported in the data-extraction table (Supplementary Table S3).

### 3.3. Methodological Quality

The methodological quality of the included studies, appraised with the Mixed Methods Appraisal Tool (MMAT, Hong et al., 2018), is presented per criterion in the scoring grid (Table 2), with the MMAT criteria for each design category defined in the accompanying key (Table 3). In line with the developers’ guidance, the individual criterion ratings were not combined into a single overall score, and we therefore do not summarise study quality with a global label; instead, the most common limitations across studies concerned small and selective samples, incomplete adjustment for confounding, and, for observational and scale-based studies, the absence of blinding and possible non-response bias. The two scale-development and validation studies (Bieleke et al., 2025; Cheval et al., 2024) were appraised under the MMAT category for quantitative descriptive studies, whose criteria (sampling relevance, sample representativeness, appropriateness of measurements, low risk of non-response bias, and appropriateness of the statistical analysis) map directly onto the design of a psychometric validation study; we judged this a better fit than the randomised or non-randomised categories, which presuppose an intervention or exposure contrast absent from a validation design. No study was excluded on the basis of methodological quality.

### 3.4. Synthesis of Findings

Owing to the heterogeneity in designs, populations, and measures, the findings are synthesised narratively. They are organised by the direction of the relationship examined and, within each direction, by the effort construct and the nature of the activity measure; neural correlates and the ecological reach of the evidence are then drawn together.

#### 3.4.1. Does Effort Valuation Predict Physical Activity?

Seven studies examined whether the way individuals value effort relates to how active they are. In a pair of experiments, Harris and Bray (2019, 2021) showed that, when young adults chose between a bout of moderate-to-vigorous exercise and a sedentary alternative, their decisions were shaped by anticipated effort and by an explicit weighing of benefits against costs: higher cost valuations were associated with a lower likelihood of choosing to exercise, and subjective benefit–cost evaluations mediated the influence of mental fatigue upon that choice. Extending this into daily life, Harris and Bray (2023) paired ecological momentary assessment with accelerometry and found that, within individuals, moments of higher benefit-versus-cost valuation were associated with more moderate-to-vigorous physical activity, whereas momentary mental fatigue predicted less—the first demonstration of these effects beyond the laboratory. In a clinically relevant sample, Bernacer et al. (2025) reported that adults with more pronounced ADHD symptoms showed steeper physical effort discounting and, in parallel, more sedentary and less healthy lifestyles. In the largest such study, Bieleke et al. (2025) developed and validated the Value of Physical Effort scale and showed, in a longitudinal design, that a greater valuation of physical effort forecast subsequent physical activity and exercise behaviour. In a complementary scale-validation, Cheval et al. (2024) developed the Physical Effort Scale and found that approach and avoidance tendencies toward physical effort explained individuals’ usual physical-activity levels. Turning to the structure of the choice itself, Taylor et al. (2026) asked participants to choose between exercise scenarios differing in the duration and intensity of effort, showing that effort duration was generally more costly than intensity, with autonomous motivation reshaping how these costs were weighed across both hypothetical and consequential choices.

#### 3.4.2. Does Exercise Reshape Effort Valuation?

Three studies approached the relationship from the opposite direction, asking whether exercise itself alters the valuation of effort. Bernacer et al. (2019) followed sedentary adults across a supervised fitness programme (an uncontrolled, longitudinal pre–post design) and observed that coupling within an amygdala–cingulate network during effort-based decisions strengthened as participants moved toward an active lifestyle, an effect most pronounced in those with lower baseline effort sensitivity. In an experimental test, Wardle et al. (2018), in a within-subject randomized crossover, found that a single acute bout of exercise increased participants’ willingness to expend effort for reward relative to a sedentary control session, with general fitness exerting no comparable moderating effect. Complementing these, Chong et al. (2018), in a cross-sectional comparison, compared elite endurance athletes with matched non-athletes and found that, although athletes were willing to exert greater physical effort, the two groups displayed qualitatively distinct patterns of effort discounting, suggesting that sustained training is accompanied by a reshaped, and not merely heightened, valuation of effort.

#### 3.4.3. Effort-Based Decision-Making in Clinical Populations

Two of the included studies situated effort valuation within clinical groups. Beyond the ADHD findings of Bernacer et al. (2025) noted above, Colón-Semenza et al. (2021) administered an effort-based decision-making task framed explicitly around exercise to people with Parkinson’s disease and to matched controls; patients valued effort and reward broadly similarly to controls, and components of motivation predicted their physical-effort decisions, pointing to motivation, rather than effort-cost sensitivity in itself, as a candidate lever for promoting activity in this population.

#### 3.4.4. Neural and Physiological Correlates

Three studies offered a window onto the neural and physiological underpinnings of the relationship. Using functional magnetic resonance imaging, Bernacer et al. (2019) localised the exercise-related change in effort valuation to an amygdala–cingulate network; with electroencephalography, Renoud-Grappin et al. (2025) demonstrated that event-related potential indices of effort-based motivation can be captured in real time during cycling, establishing the feasibility of monitoring motivation during genuine exercise; and the computational modelling performed by Chong et al. (2018) dissociated the valuation of physical from cognitive effort. Together, these findings suggest that the neural signatures of effort valuation are both sensitive to physical activity and, in principle, measurable during it, though the evidence remains sparse and methodologically diverse.

#### 3.4.5. Ecological Validity and the Two Tiers

Finally, a striking feature of the evidence base concerns its ecological texture. Eleven of the twelve included studies fell within Tier 1, relating effort valuation to exercise that was genuinely performed, experimentally manipulated, or reflected in athletic training status. Only one study, Taylor et al. (2026), reached into Tier 2, and even then only partially, pairing hypothetical exercise choices with a consequential, performed condition. This near-absence of Tier 2 is itself a finding: until very recently the literature connected effort-based decision-making to exercise almost exclusively through real activity, and the controlled, simulated exercise decisions familiar from adjacent neuroeconomic research have only just begun, with Taylor et al. (2026) to be explored in this context.

## 4. Discussion

### 4.1. Summary of Principal Findings

This review set out to map a deceptively simple question—whether the brain’s valuation of physical effort is bound up with how much people actually move—and, in doing so, to address a piece of the neuroeconomic puzzle that has been neglected. Across twelve eligible studies, a coherent, if still-emerging, picture took shape. Effort valuation and physical activity appear to be associated in both of the hypothesised directions: the subjective cost a person assigns to effort helps to predict whether they choose to be active, and being active, in turn, appears to reshape how costly effort comes to feel. Tellingly, the studies that captured this did so almost entirely through real, enacted exercise rather than abstract proxies, lending the evidence an ecological authenticity that is rare in the wider effort-valuation literature—even as the controlled, simulated paradigms of neuroeconomics have only very recently begun to appear in this context.

### 4.2. Effort: The Overlooked Cost, and a Reciprocal Architecture

Perhaps the most telling observation concerns which cost the field has, until now, chosen to study. Where decision science has been brought to bear on physical activity, it has leaned heavily on delay discounting, the devaluation of rewards that are merely distant in time (Tate et al., 2015; Sofis et al., 2017; Berardi et al., 2024). Yet, exercise is defined less by the remoteness of its rewards than by the effort it demands here and now; effort is the cost paid in the body, in the present moment, with every step and every repetition. This automatic pull away from exertion is precisely what the Theory of Effort Minimization in Physical Activity (TEMPA; Cheval & Boisgontier, 2021) places at the centre of the inactivity pandemic, casting the attraction to effort minimization as a fundamental, and long-overlooked, driver of behaviour. That the effort construct, most intrinsic to exercise, should have remained comparatively unexamined is itself an important and under-explored gap, and it is the one this review brings into focus.

**Figure 2:**
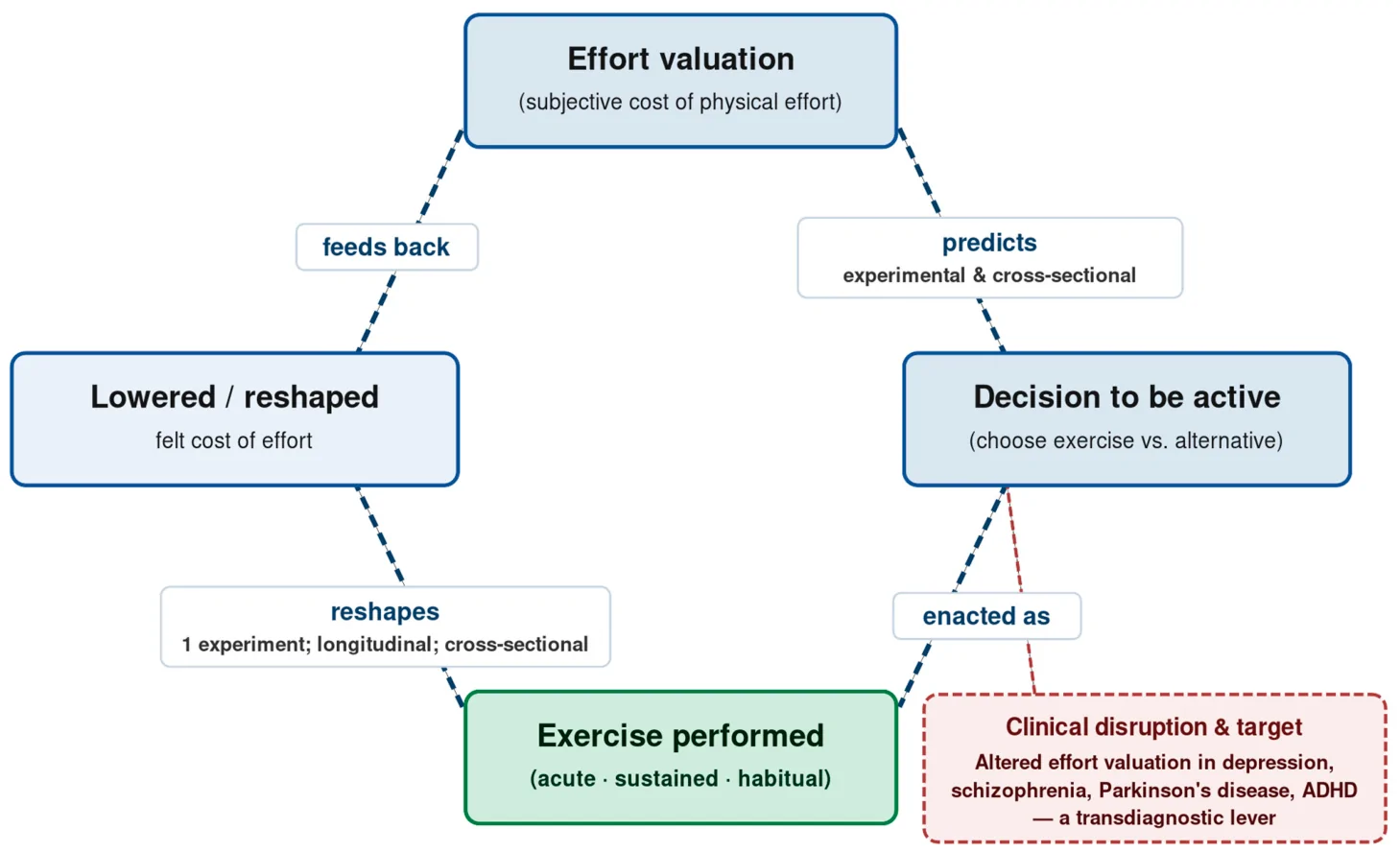
A reciprocal model of effort valuation and physical activity. The blue dashed arrows denote the hypothesised reciprocal effort–activity loop synthesised in this review (arrows are dashed to signal hypothesised, not established, pathways), with the evidence type supporting each directional pathway indicated (experimental and cross-sectional for effort valuation → activity; one experiment, one longitudinal, and cross-sectional studies for exercise → effort valuation): the subjective cost of effort shapes the decision to be active, which is enacted as exercise, which in turn appears to lower or reshape the felt cost of effort. The red dashed element marks clinical conditions in which effort valuation is altered and which may be targeted through the loop. Tier 1 = real, enacted exercise (most evidence); Tier 2 = simulated/hypothetical exercise decisions (emerging; Taylor et al., 2026). Because most contributing evidence is cross-sectional or uncontrolled, the loop is presented as an integrative hypothesis rather than an established causal mechanism.

The shape of the evidence suggests, further, that effort valuation and activity are linked in both directions rather than in one. On one side, individuals who discount effort less steeply, or who weigh its costs less heavily against its benefits, are more inclined to choose activity (Harris & Bray, 2019, 2021, 2023; Bernacer et al., 2025). On the other, the experience of exercising, whether a single acute bout (Wardle et al., 2018), a sustained programme that turns the sedentary active (Bernacer et al., 2019), or the accumulated training of the athlete (Chong et al., 2018), appears to lower or reconfigure the felt cost of effort. Read together, these strands hint at a self-reinforcing loop (Figure 2) in which becoming active makes effort feel less costly, which in turn makes further activity more likely. Such a loop would carry real significance, for it implies that the very barrier to exercise, the aversiveness of effort, is not fixed but malleable, and may yield, in part, to the behaviour it seems to obstruct. Importantly, because most of the contributing evidence is cross-sectional, this reciprocal loop is best read as an integrative hypothesis that the field is now positioned to test directly, rather than as an established causal mechanism. The two directions are, moreover, unevenly evidenced: the proposition that exercise reshapes effort valuation rests on a single within-subject experiment (Wardle et al., 2018) together with one uncontrolled longitudinal study (Bernacer et al., 2019) and a cross-sectional athlete comparison (Chong et al., 2018), whereas the proposition that effort valuation shapes activity draws on experimental manipulations of effort in exercise choice (Harris & Bray, 2019, 2021; Taylor et al., 2026) alongside predominantly cross-sectional, self-report associations (Bernacer et al., 2025; Bieleke et al., 2025; Cheval et al., 2024). Only the experimental studies license causal statements, and even these concern momentary choices rather than habitual activity.

### 4.3. An Ecologically Grounded Yet Mechanistically Sparse Field

At the same time, the review exposes a curious asymmetry in the field’s methodological design. The included studies are, almost without exception, ecologically rich: they tether effort valuation to genuine activity: accelerometer-measured movement in daily life, supervised fitness programmes, real cycling, the training histories of athletes. Only one, the very recent forced-choice study of Taylor et al. (2026), has begun to populate Tier 2, and even then by pairing hypothetical exercise choices with a consequential, performed condition. In one sense this is a strength, for the evidence speaks to exercise as it is actually lived rather than merely as it is modelled. This should not, however, be equated with ecological validity: ‘enacted’ does not entail ‘naturalistic.’ A controlled laboratory cycling task (Tier 1c), though performed, samples everyday activity less faithfully than device-measured behaviour in daily life (Tier 1a), and a hypothetical choice embedded in a naturalistic setting could in principle predict long-term activity as well as, or better than, an artificial laboratory task. Ecological validity is multidimensional, and our tiers index the enacted–simulated distinction rather than ecological validity per se. In another, it is a limitation, for the controlled, parametric paradigms that have allowed neuroeconomics to isolate the computations behind effort-based choice (Klein-Flügge et al., 2015; Rigoli & Pezzulo, 2022; Bogdanov & Pizzagalli, 2026) have scarcely been turned toward exercise. The field is thus, somewhat paradoxically, ecologically grounded yet mechanistically thin: it can show that effort valuation and activity move together, but it is not yet well placed to explain precisely how. Carrying the rigour of the laboratory into the texture of real movement, and the authenticity of real movement back into the laboratory, is perhaps the central design challenge that lies ahead.

Two studies illustrate just how close, and yet how far, this near-empty tier remains. Studer et al. (2019) and Van As et al. (2021) both framed their paradigms explicitly around exercise and the willingness to undertake effortful, health-relevant action, yet in each the effort was exerted through a laboratory proxy: a precommitment task in the former, a handgrip manipulation in the latter, rather than through exercise genuinely performed. Precisely because we held consistently to a real-environment criterion, requiring that exercise be enacted rather than merely invoked, these otherwise pertinent studies fell just outside eligibility; that very boundary is itself a measure of how sparsely the simulated middle ground has so far been cultivated. A parallel line of work has built simulated exercise-choice paradigms, for instance the behavioural-alternatives and DPEX tasks of Timme and Brand (2024) and Timme et al. (2023), yet these frame the choice through affect and preference rather than effort, so that the specifically effort-based simulation of exercise decisions, of which Taylor et al. (2026) is the first instance, remains almost wholly unexplored.

### 4.4. Clinical and Translational Implications

These findings also carry potential clinical relevance. A diminished willingness to invest effort sits close to the heart of the motivational disturbances seen across depression, schizophrenia, Parkinson’s disease, anorexia nervosa, ADHD, and the aftermath of stroke (Hird et al., 2024; Décombe et al., 2020; King et al., 2025; Erfanian Abdoust et al., 2024), conditions in which physical inactivity is also stubbornly common. The two included clinical studies are instructive. In Parkinson’s disease, effort and reward were valued much as in healthy controls, with motivation rather than effort-cost sensitivity predicting physical-effort decisions (Colón-Semenza et al., 2021); in adults with ADHD symptoms, steeper effort discounting accompanied more sedentary lives (Bernacer et al., 2025). Together they raise the possibility that effort-based decision-making is a transdiagnostic lever, a shared mechanism through which inactivity becomes entrenched, and therefore a shared target through which it might be eased. If the reciprocal loop described above holds, interventions that gently lower the subjective cost of effort, or that harness the cost-reducing effect of early activity, may prove especially valuable precisely where motivation is most fragile. These clinical implications must, however, be regarded as tentative: only two of the included studies examined clinical or symptom-based populations (Parkinson’s disease and adult ADHD), and the broader transdiagnostic framing draws on the background literature that was not part of the systematically searched evidence. The clinical potential of effort-based decision-making is therefore best treated as a hypothesis to be tested in adequately powered clinical studies rather than as an established application.

### 4.5. Implications for Theory and Practice

For theory, these findings invite a closer integration of two areas of research that have largely developed apart. Dominant models of exercise behaviour rest largely upon attitudes, intentions, and self-efficacy (André et al., 2024), and seldom incorporate the formal valuation of cost and benefit that neuroeconomics makes explicit. Embedding an effort-cost computation within these frameworks, treating the decision to move as an act of valuation, and not merely of intention, may help to explain the stubborn gap between what people intend and what they ultimately do. Recent integrative syntheses of the psychophysiological foundations of physical-activity behaviour (Gerber et al., 2025) issue a similar call, urging that motivational and neuroscientific accounts of movement be drawn together rather than pursued in parallel. For practice, the implication is more hopeful than fatalistic: if the felt cost of effort can be reshaped, then strategies that render movement less effortful or more immediately rewarding, gradual habituation, affect-centred rather than health-centred framing (Maltagliati et al., 2024; Cheval et al., 2025), and the early, deliberate cultivation of activity habits, may matter as much as any exhortation about distant health benefits.

### 4.6. Strengths and Limitations

This review has several strengths. It was conducted against a pre-registered protocol, screened and appraised in duplicate by independent reviewer groups, reported in accordance with the PRISMA 2020 statement, and built upon explicit, theory-driven eligibility criteria, the two ‘gates’ of an effort-based decision-making construct and a genuine exercise outcome, together with a framework examining both hypothesised directions and the enacted–simulated dimension, which allowed the ecological reach of the evidence to be examined directly. A comprehensive multi-database search was reinforced by backward and forward citation searching.

Its limitations must be acknowledged with equal candour. The eligible evidence is small—twelve studies—and, while this sparseness is itself the central finding, it necessarily tempers the strength of any conclusion. In addition, 31 of the 123 reports sought (approximately one quarter) could not be retrieved, their full texts lying behind subscription barriers not covered by our institutional access despite our attempts. Although citation searching helped offset these unretrieved reports, this non-retrieval rate raises the possibility of availability bias—whereby harder-to-obtain reports may systematically differ from those retrieved—which we acknowledge as a limitation. A per-record list of the 31 unretrieved reports was not retained; we state this transparently and acknowledge it, together with the resulting possibility of availability bias, as a limitation of the review process. The restriction to English-language articles may have introduced a measure of language bias. Finally, the included studies were themselves heterogeneous in design, population, activity measure, and—importantly—in the effort construct itself and how it was measured, ranging from behavioural decision and discounting tasks to self-report valuation scales and neural indices, which do not necessarily capture equivalent phenomena, precluding meta-analysis, and many were cross-sectional, limiting causal inference about the very directionality this review sought to characterise. Relatedly, the neural mechanisms of effort valuation were not the object of our systematic search—they are summarised from dedicated reviews as background—and only a minority of the included studies incorporated neural or physiological measures; inferences about neural substrates are therefore preliminary. A further caveat concerns common-method variance: in the two scale-validation studies (Bieleke et al., 2025; Cheval et al., 2024), both the effort-valuation exposure (the VoPE and PES scales) and physical activity were assessed by self-report, so their associations may be inflated by common-method variance, social desirability, generally positive self-evaluation, or exercise identity, rather than reflecting an independent effect of effort valuation on enacted activity.

### 4.7. Certainty of the Evidence and Reporting Bias

Given the small number of studies, their heterogeneity, and the predominance of observational and cross-sectional designs, the overall certainty of the evidence is low. We did not undertake a formal GRADE assessment, as the diversity of designs, constructs, and outcomes precluded a meaningful pooled estimate; instead, confidence in each of the two hypothesised directions is limited by the same features that constrain causal inference—small and often selective samples (several with N < 100), single-bout or single-session designs, and heterogeneous effort and activity measures. Formal statistical evaluation of publication bias (for example, funnel-plot asymmetry) was not feasible with so few, methodologically diverse studies; nonetheless, the risk of reporting and publication bias should be regarded as non-trivial, since small studies reporting significant or positive associations are more likely to be published and cited, which may inflate the apparent strength of the effort–activity relationship. Two observations partly temper this concern: the evidence base includes at least one clearly null finding (no difference in effort-based decisions between people with Parkinson’s disease and controls; Colón-Semenza et al., 2021), and several associations derive from pre-registered or comparatively large-sample studies. Selective outcome reporting within studies cannot be excluded and was not separately assessed. Taken together, these considerations reinforce our framing of the reciprocal model as hypothesis-generating and indicate that the findings should be interpreted with caution, pending larger, pre-registered, and methodologically standardised research.

### 4.8. Directions

The path forward follows naturally from these gaps. Most pressing is the need for studies that measure effort-based decision-making and device-assessed, real-world physical activity within the same individuals, and that do so longitudinally, so that the reciprocal loop may be tested rather than inferred. The controlled paradigms of neuroeconomics should be deliberately extended to exercise, including the simulated and hypothetical exercise decisions of our empty Tier 2—so that mechanism can be isolated with the precision the laboratory affords and then validated against genuine movement. The portable neural and physiological monitoring foreshadowed by Renoud-Grappin et al. (2025) offers a promising means of observing effort valuation as it unfolds during real activity. Greater standardisation of effort tasks and activity measures would allow future findings to be pooled with confidence. And, given the clinical stakes, intervention studies should test directly whether lowering the subjective cost of effort can increase physical activity in the populations where inactivity exacts its heaviest toll.

## 5. Conclusions

Physical inactivity is a major and persistent behavioural and public-health challenge, and this review suggests that part of its remedy may lie in how the brain values effort. Across a small but coherent body of evidence, the subjective cost of effort and the behaviour of exercising appear to be associated in both hypothesised directions—through real and lived activity rather than abstract choice—although, given the largely observational evidence, this reciprocity remains a hypothesis to be tested rather than an established mechanism. Effort-based decision-making thus emerges as a real and neglected piece of the neuroeconomic puzzle of why we move, or fail to move. The field is young, its evidence still sparse, and its mechanistic foundations only partly laid; yet, the questions it opens are precise, tractable, and worth pursuing. In naming this gap and giving it shape, we hope to have offered a clear starting point and agenda for future research.

## Figures and Tables

**Table 1:** Characteristics of Included Studies.

Study	Country	Design & Sample	Effort Construct/Task	Exercise/PA Measure	Direction	Tier	Key Finding
Bernacer et al. (2019)	Spain	Longitudinal pre/post, fMRI; 19 sedentary adults	Effort discounting/subjective value (amygdala–cingulate fMRI)	Supervised fitness programme (sedentary → active)	PA → effort	1	Amygdala–cingulate coupling during effort decisions strengthened after becoming active, most in those with low effort sensitivity.
Bernacer et al. (2025)	Spain	Cross-sectional; 181 adults (18–33 y)	Physical effort discounting (EEfRT + hypothetical task)	Self-reported PA & sedentary behaviour	Assoc.	1	More ADHD symptoms → steeper effort discounting and more sedentary, unhealthier lifestyles.
Bieleke et al. (2025)	Germany	Scale development & validation; 3 studies, N = 1267 (incl. longitudinal)	VoPE self-report scale (valuation of physical effort)	Self-reported sport/exercise & PA (longitudinal)	Effort → PA	1	VoPE is reliable and valid and longitudinally forecasts PA and sport/exercise behaviour.
Chong et al. (2018)	UK	Cross-sectional, computational modelling; 20 elite rowers vs. 20 matched non-athletes	Cognitive & physical effort discounting (subjective value)	Athletic training status (elite endurance training)	assoc.	1	Athletes willing to exert more physical effort; distinct (concave vs. convex) effort-discounting patterns vs. non-athletes.
Cheval et al. (2024)	France/Switzerland	Scale development & validation; 3 studies (Study 3 *n* = 297)	PES: approach/avoidance toward physical effort (self-report)	Self-reported usual PA level	Effort → PA	1	PES (approach/avoidance) is reliable and valid and explained usual PA; grounded in TEMPA.
Colón-Semenza et al. (2021)	USA	Cross-sectional case–control; 32 Parkinson’s patients vs. 23 controls	Effort-based decisions for exercise (physical effort task)	Cycling-based physical effort task	Assoc.	1	Parkinson’s patients valued effort/reward like controls; motivation components predicted their effort decisions.
Harris and Bray (2019)	Canada	Randomised lab experiment; N = 55 young adults	Mental fatigue → benefit–cost valuation	Choice of a 22 min MVPA bout (enacted)	Effort → PA	1	Higher cost valuations → less likely to exercise; benefit–cost valuation mediated the fatigue–exercise link.
Harris and Bray (2021)	Canada	Mixed-methods randomised experiment; N = 84 young adults	Anticipated effort & benefit–cost valuation	Choice of a 20 min MVPA bout (enacted)	Effort → PA	1	Anticipated effort and subjective valuations predicted choosing to exercise vs. a sedentary option.
Harris and Bray (2023)	Canada	Observational EMA + accelerometer, 7 days; 100 young adults	Momentary benefit–cost valuations & mental fatigue	Device-measured MVPA in daily life	Effort → PA	1	First real-world evidence: higher benefit-vs-cost → more MVPA; momentary mental fatigue → less.
Renoud-Grappin et al. (2025)	France	Feasibility experiment, EEG/ERP; 20 participants	Effort-based decisions (EEfRT) during exercise	Cycling with concurrent ERP	Assoc.	1	Feasible to monitor effort-based motivation (ERP) in real time during cycling.
Taylor et al. (2026)	UK	Three forced-choice studies (24 choices); Studies 1–2 hypothetical, Study 3 consequential	Forced choices between exercise scenarios varying effort duration/intensity	Hypothetical (Tier 2) & performed (Tier 1) choices	Effort → PA	1 & 2	Effort duration costlier than intensity; autonomous motivation shifts effort’s value; hypothetical vs. consequential choices differ.
Wardle et al. (2018)	USA	Within-subject randomised (exercise vs. sedentary); 35 healthy adults	Reward/effort functioning (EEfRT)	Acute exercise bout (manipulated)	PA → effort	1	An acute exercise bout raised willingness to expend effort for reward; fitness did not moderate.

Legend: PA, physical activity; fMRI, functional magnetic resonance imaging; EEfRT, Effort-Expenditure for Rewards Task; MVPA, moderate-to-vigorous physical activity; EMA, ecological momentary assessment; EEG, electroencephalography; ERP, event-related potential; ADHD, attention-deficit/hyperactivity disorder; PD, Parkinson’s disease; PES, Physical Effort Scale; VoPE, Value of Physical Effort scale; TEMPA, Theory of Effort Minimization in Physical Activity. Direction denotes the relationship examined (Effort → PA, effort valuation predicting activity; PA → effort, exercise reshaping effort valuation; Assoc., association only). Tier 1 = real, enacted exercise; Tier 2 = simulated/hypothetical exercise decisions. Numerical effect estimates and measures of precision for each study are reported in Supplementary Table S3.

**Table 2:** Scoring grid (MMAT, Mixed Methods Appraisal Tool, Hong et al., 2018).

Study	Design/MMAT Category	SQ1	SQ2	C1	C2	C3	C4	C5	Brief Rationale/Notes
Bernacer et al. (2019)	Category 3—Quantitative non-randomized; single-group pre–post fitness intervention	Y	Y	CT	Y	N	N	Y	Small selected sample; appropriate behavioural/fMRI measures; attrition; no control group; adherence monitored.
Bernacer et al. (2025)	Category 4—Quantitative descriptive; cross-sectional association study	Y	Y	Y	CT	Y	CT	Y	Relevant sample and measures; representativeness unclear; missingness/nonresponse bias unclear; analyses appropriate.
Bieleke et al. (2025)	Category 4—Quantitative descriptive; scale validation	Y	Y	Y	CT	Y	CT	Y	C2/C4 rated CT—verify sampling & attrition.
Cheval et al. (2024)	Category 4—Quantitative descriptive; scale validation	Y	Y	Y	CT	Y	CT	Y	C2/C4 rated CT—verify sampling & nonresponse.
Chong et al. (2018)	Category 3—Quantitative non-randomized; elite rowers vs. matched controls	Y	Y	CT	Y	Y	Y	Y	Narrow Oxford rower/control sample; appropriate effort tasks; complete final data; matching/confound checks reported.
Colón-Semenza et al. (2021)	Category 3—Quantitative non-randomized; PD vs. healthy control comparison	Y	Y	N	Y	Y	Y	Y	Convenience sample with mild–moderate/active PD limits generalizability; measures appropriate; analyses adjusted for age, walking capacity and motor severity.
Harris and Bray (2019)	Category 2—Quantitative randomized controlled experiment; high vs. low cognitive demand	Y	Y	CT	Y	Y	Y	Y	Randomization reported but sequence generation unclear; baseline exercise variables comparable; blinded researcher; manipulation checks supported adherence.
Harris and Bray (2021)	Category 5—Mixed methods; randomized experiment plus semi-structured interviews	Y	Y	Y	Y	Y	Y	Y	Clear rationale, integration, interpretation and handling of divergence; quantitative and qualitative components adequately reported.
Harris and Bray (2023)	Category 4—Quantitative descriptive; EMA + accelerometry observational study	Y	Y	Y	N	Y	N	Y	Relevant EMA sampling, but young predominantly female/active sample; incomplete prompts linked to age, sex and time of day; multilevel analyses appropriate.
Renoud-Grappin et al. (2025)	Category 4—Quantitative randomized crossover feasibility experiment; cycling vs. no-cycling	Y	Y	CT	Y	Y	CT	Y	Randomized session order reported but method unclear; crossover design; EEG/objective outcomes; assessor blinding not reported; cycling protocol monitored.
Taylor et al. (2026)	Category 3—Quantitative non-randomized; forced-choice experiment	Y	Y	CT	Y	Y	Y	Y	C1 rated CT—verify representativeness.
Wardle et al. (2018)	Category 2—Quantitative randomized crossover experiment; acute running vs. sedentary rest	Y	Y	CT	Y	Y	N	Y	Randomized counterbalanced order reported but method unclear; within-subject design; not blinded/no placebo exercise; exercise dose standardized.

Legend: Y = Yes; N = No; CT = Cannot tell. SQ1–SQ2, the two screening questions common to all designs; C1–C5, the five criteria of each study’s design category (see Table 3). Per MMAT guidance, ratings are reported per criterion and not summed into an overall score.

**Table 3:** Criterion key by MMAT category.

MMAT Category	Criteria Represented by C1–C5 in the Scoring Grid
Category 2—Quantitative randomized controlled trials	2.1 Randomization appropriately performed; 2.2 Groups comparable at baseline; 2.3 Complete outcome data; 2.4 Outcome assessors blinded; 2.5 Participants adhered to assigned intervention.
Category 3—Quantitative non-randomized studies	3.1 Participants representative of target population; 3.2 Measurements appropriate; 3.3 Complete outcome data; 3.4 Confounders accounted for; 3.5 Intervention/exposure administered as intended.
Category 4—Quantitative descriptive studies	4.1 Sampling strategy relevant; 4.2 Sample representative; 4.3 Measurements appropriate; 4.4 Low risk of nonresponse bias; 4.5 Statistical analysis appropriate.
Category 5—Mixed-methods studies	5.1 Adequate rationale for mixed methods; 5.2 Effective integration of components; 5.3 Outputs interpreted together; 5.4 Divergences/inconsistencies addressed; 5.5 Components adhere to quality criteria of each tradition.

## Data Availability

This is a systematic review; all data supporting its findings derive from the published studies cited. The complete data-extraction table is provided as Supplementary Table S3, and the full search strategies and citation-search tracker are provided as Supplementary Tables S1 and S2, respectively. No new primary data were generated. Further details are available from the corresponding author upon reasonable request.

## Supplementary Materials

The following supporting information can be downloaded at https://www.mdpi.com/article/10.3390/bs16091508/s1. Table S1: Complete Search strategies; Table S2: Citation-Search Tracker (Backward & Forward); Table S3: Full Data-Extraction Table.

## Abbreviations

The following abbreviations are used in this manuscript:

ADHD	Attention-Deficit/Hyperactivity Disorder
DPEX	Decisional Preferences in Exercising
EEfRT	Effort-Expenditure for Rewards Task
EEG	Electroencephalography
EMA	Ecological Momentary Assessment
ERP	Event-Related Potential
fMRI	Functional Magnetic Resonance Imaging
INPLASY	International Platform of Registered Systematic Review and Meta-Analysis Protocols
MMAT	Mixed Methods Appraisal Tool
MVPA	Moderate-to-Vigorous Physical Activity
PA	Physical Activity
PD	Parkinson’s Disease
PECO	Population, Exposure, Comparator, Outcome
PES	Physical Effort Scale
PRISMA	Preferred Reporting Items for Systematic Reviews and Meta-Analyses
TEMPA	Theory of Effort Minimization in Physical Activity
vmPFC	Ventromedial Prefrontal Cortex
VoPE	Value of Physical Effort
