# Supplementary material for: A Piece of the Neuroeconomic Puzzle: Effort-Based Decision-Making and Physical Activity—A Systematic Review
Source: Behav Sci (Basel). 2026 Aug 27;16(9):1508. doi: 10.3390/bs16091508 (PMC13603856; doi:10.3390/bs16091508)
Supplement: Supplementary file 1 [file behavsci-16-01508-s001.zip › Supplementary Table S1-search strategy.pdf]

Supplementary Table S1. Complete search strategies

Systematic review:

"A Piece of the Neuroeconomic Puzzle: Effort-Based Decision-Making and Physical Activity — A Systematic Review."

All database searches were executed in June 2026, from each database's inception to that date, with no lower date limit. (Update statement: the database searches were not updated between the original search and manuscript submission)

1. Scopus

Platform: Scopus (Elsevier). Search field: TITLE-ABS-KEY (title, abstract, author and index keywords). Date searched: 15 June 2026.

Filters applied: Document type = Article and Review; Language = English; no date limit.

Records retrieved: 286 (raw) → 258 after filters.

TITLE-ABS-KEY (

("effort-based decision*" OR "effort-based choice" OR "effort discount*"

OR "effort cost*" OR "cost of effort" OR "effort expenditure"

OR "effort allocation" OR "effort-related decision*" OR neuroeconomic*)

AND

(exercise OR "physical activity" OR "physical exercise" OR "exercise training"

OR "aerobic training" OR "resistance training" OR "physical fitness"

OR sport* OR "exercise adherence" OR "physical inactivity"

OR "sedentary behav*" OR walking OR running))

2. Web of Science Core Collection

Platform: Web of Science (Clarivate). Search field: Topic (TS = title, abstract, author keywords, Keywords Plus). Date searched: 15 June 2026.

Filters applied: Document type = Article and Review; Language = English; no date limit.

Records retrieved: 199 (raw) → 159 after filters.

TS=(("effort-based decision*" OR "effort-based choice" OR "effort discount*"

OR "effort cost*" OR "cost of effort" OR "effort expenditure"

OR "effort allocation" OR "effort-related decision*" OR neuroeconomic*)

AND (exercise OR "physical activity" OR "physical exercise" OR "exercise training"

OR "aerobic training" OR "resistance training" OR "physical fitness"

OR sport* OR "exercise adherence" OR "physical inactivity"

OR "sedentary behav*" OR walking OR running))

3. PubMed / MEDLINE

Platform: PubMed (U.S. National Library of Medicine). Search fields: [tiab] (title/abstract) plus Medical Subject Headings [Mesh]. Date searched: 15 June 2026.

Filters applied: article-type limits (Books and Documents excluded); no date limit; non-human (animal) records removed at screening.

Records retrieved: 69 (raw) → 68 after filters.

("effort-based decision making"[tiab] OR "effort-based decisions"[tiab]

OR "effort-based choice"[tiab] OR "effort discounting"[tiab]

OR "effort cost"[tiab] OR "effort costs"[tiab] OR "cost of effort"[tiab]

OR "effort expenditure"[tiab] OR "effort allocation"[tiab]

OR "effort-related decision making"[tiab] OR neuroeconomic*[tiab])

AND

("Exercise"[Mesh] OR exercise[tiab] OR "physical activity"[tiab]

OR "physical exercise"[tiab] OR "exercise training"[tiab]

OR "aerobic training"[tiab] OR "resistance training"[tiab]

OR "physical fitness"[tiab] OR "Physical Fitness"[Mesh] OR sport[tiab]

OR sports[tiab] OR "exercise adherence"[tiab] OR "physical inactivity"[tiab]

OR "Sedentary Behavior"[Mesh] OR "sedentary behavior"[tiab]

OR "sedentary behaviour"[tiab] OR walking[tiab] OR running[tiab])

4. Records identified and citation searching

Total records identified across databases (after filters): 258 + 159 + 68 = 485. Removal before screening, screening, and full-text assessment are reported in the PRISMA 2020 flow diagram (Figure 1).

Delay/temporal discounting terms were deliberately excluded as search constructs (treated as background) to keep the review centred on the effort cost; a neural-mechanism block was not ANDed in, as it would have excluded the behavioural studies central to the question.

Citation searching. Backward searching (reference lists of the included studies and of key reviews) and forward searching ("Cited by" via Google Scholar, Scopus, and Web of Science) of the included studies were conducted in June 2026 [final forward searches completed on 28 June]. Citation searching identified three additional eligible studies (Bieleke et al., 2025; Cheval, Maltagliati et al., 2024; Taylor et al., 2026). A full citation-search tracker is available on request / as Supplementary Table S2.
