# Supplementary material for: A Piece of the Neuroeconomic Puzzle: Effort-Based Decision-Making and Physical Activity—A Systematic Review
Source: Behav Sci (Basel). 2026 Aug 27;16(9):1508. doi: 10.3390/bs16091508 (PMC13603856; doi:10.3390/bs16091508)
Supplement: Supplementary file 1 [file behavsci-16-01508-s001.zip › Supplementary Table S2 - Citation search tracker.pdf]

Supplementary Table S2. Citation-Search Tracker (Backward & Forward)

Backward and forward citation searching are complete for all included studies. Three additional eligible studies were identified and included (Bieleke et al., 2025; Taylor et al., 2026; Cheval, Maltagliati et al., 2024 (PES)), n = 12. Other candidates (Brown & Bray, 2019; Jonker, 2010; and various performance / affect / reverse-direction studies) were assessed and retained as background

A. Source studies searched (backward + forward)

Included study (search source)	Backward (reference list)	Forward ('Cited by')
Bernacer et al. 2019	Done — none eligible	Done — none eligible
Bernacer et al. 2025	Done — none eligible	Done (8 citations) — none eligible
Chong et al. 2018	Done — none eligible	Done — none eligible
Harris & Bray 2019	Done — none eligible	Done — none eligible
Harris & Bray 2021	Done — none eligible	Done — none eligible
Harris & Bray 2023	Done — none eligible	Done — none eligible
Renoud-Grappin et al. 2025	Done — none eligible	No citing articles yet
Wardle et al. 2018	Done — none eligible	Done — none eligible
Colón-Semenza et al. 2021	Done — none eligible	Done — none eligible
Bieleke et al. 2025	Done — none eligible	Done - Taylor 2026 + PES (Cheval/Maltagliati 2024) INCLUDED

B. Studies added through citation searching (now included)

Study	Why eligible	Tier	Found via
Bieleke et al. (2025)	Value of Physical Effort (VoPE) scale; longitudinally forecasts PA	Tier 1	Backward (from Stähler review)
Taylor et al. (2026)	Effort-based forced choices between exercise scenarios (effort duration/intensity)	Tier 1 & 2 (first Tier-2 study)	Forward (from Bieleke 2025)
Cheval, Maltagliati et al. (2024)	Physical Effort Scale (PES); approach/avoidance of effort explains usual PA	Tier 1	Forward (from Bieleke 2025)

C. Candidates assessed and excluded (background)

Candidate(s)	Reason — background, not included
Brown & Bray (2019)	Effort regulation / RPE during exercise - not a decision construct (gate 1)
Jonker et al. (2010)	Self-regulation of learning / academic outcome - off-construct
Timme & Brand (2023, 2024; DPEX)	Simulated exercise choice, but affect/preference-based, not effort - Discussion citation
Greenhouse-Tucknott (2025); Morris (2022)	Lab effort task / resting-state predictor - no real-exercise decision construct
Wellings (2025); Filipas (2021); Holgado (2025); Bray & Harris (2025)	Performance / mental-fatigue / goal-conflict - no effort-valuation construct

D. Background / theory citations gathered (for Introduction & Discussion)

Citation	Use in manuscript
Cheval & Boisgontier (2021) — TEMPA	Theory of Effort Minimization in Physical Activity - core framing (Intro & Discussion 4.2)
Gerber et al. (2025)	Psychophysiological foundations of PA behaviour & motivation - Intro & Discussion 4.5
Timme & Brand (2023, 2024)	Simulated exercise-choice paradigms (affect-based) - Discussion 4.3 contrast
