# Supplementary material for: A Piece of the Neuroeconomic Puzzle: Effort-Based Decision-Making and Physical Activity—A Systematic Review
Source: Behav Sci (Basel). 2026 Aug 27;16(9):1508. doi: 10.3390/bs16091508 (PMC13603856; doi:10.3390/bs16091508)
Supplement: Supplementary file 1 [file behavsci-16-01508-s001.zip › Supplementary Table S3- data extraction.pdf]

**Supplementary Table S3**. Data extraction and principal quantitative results of the included studies

| Study | Design & sample | Effort construct / measure | PA / exercise measure | Principal quantitative result(s) | Adjustments / limitations |
|---|---|---|---|---|---|
| **Bernacer et al. 2019** | Longitudinal pre–post, fMRI; N = 19 sedentary adults becoming active | Effort discounting in a monetary choice task; subjective value (fMRI) | Supervised fitness programme (enacted) | Mixed-effects logistic choice model: effort of the pair B = −19.40 (z = 4.90, $p < .001$); effort × habit interaction B = 7.56, 95% CI 4.12–11.01, $p < .001$ (effort's weight on choice shifted with becoming active); fMRI: amygdala–cingulate/ACC cluster, $p < .05$ SVC | Single group, no control arm; N = 19 (small); uncontrolled pre–post |
| **Bernacer et al. 2025** | Cross-sectional; N = 181 adults (18–33; 58 with ADHD) | EEfRT (immediate) + hypothetical physical effort-discounting | Self-reported physical activity & sedentary behaviour | Best-fitting choice model Wald $\chi^2(10) = 623.64$, $p < .0001$; ADHD symptoms associated with steeper physical effort discounting and with more sedentary, less healthy lifestyles (associations) | Cross-sectional; self-report PA; associational only |
| **Chong et al. 2018** | Cross-sectional case–control, computational modelling; 20 elite rowers vs 20 controls | Cognitive & physical effort discounting (subjective value) | Athletic training status | Athletes accepted the higher-physical-effort option more often (82.0 ± 1.8% vs 73.8 ± 1.8%, $p = .002$); Group × Effort interaction $F(5,190) = 8.09$, $p < .001$ (qualitatively distinct discounting) | Small, selective (elite) sample; cross-sectional; training self-selected |
| **Harris & Bray 2019** | Randomised lab experiment; N = 55 young adults | Mental fatigue → benefit–cost valuation of exercising | Choice to complete a 22-min MVPA bout (enacted) | ~Half chose to exercise (n = 30); no direct effect of cognitive-load group on choice; serial mediation showed significant indirect effects via perceived fatigue and benefit–cost valuation | Lab choice; single bout; students; short time frame |
| **Harris & Bray 2021** | Mixed-methods randomised experiment; N = 84 young adults | Anticipated effort & benefit–cost valuation | Choice to complete a 20-min MVPA bout (enacted) | Serial mediation showed a significant indirect effect of mental fatigue on the choice to exercise via anticipated effort and benefit–cost valuation | Lab choice; single bout; students |
| **Harris & Bray 2023** | Observational EMA + accelerometry, 7 days; N = 100 | Momentary benefit–cost valuation & mental fatigue | Device-measured MVPA in daily life | Within-person, higher benefit-vs-cost predicted more MVPA ($p < .001$); momentary mental fatigue predicted less MVPA (p = | Observational (within-person); no manipulation; |

| | | | | | |
|---|---|---|---|---|---|
| | | | | .004); multilevel model $\chi^2(4) = 12.59$, $p = .014$ (coefficients & 95% CIs in source) | young, mostly female |
| **Renoud-Grappin et al. 2025** | Feasibility experiment, EEG/ERP; N = 20 | EEfRT administered during cycling (ERP indices) | Cycling (real exercise) with concurrent ERP | Feasibility demonstrated; ERP modulations during cycling, e.g., feedback effect $F(1,18) = 4.63$, $p = .045$, $\eta p^2 = .20$; FRN by anteriority $F(2,36) = 5.74$, $p = .007$, $\eta p^2 = .24$ | N = 20; feasibility/methods focus; not a hypothesis test of the effort–PA link |
| **Wardle et al. 2018** | Within-subject randomised crossover (exercise vs sedentary); N = 35 healthy adults | Reward/effort functioning (EEfRT) | Acute exercise bout (manipulated) | Powered to detect d = 0.53; an acute exercise bout increased motivation/willingness to expend effort for reward (EEfRT) relative to the sedentary session; general fitness did not moderate | N = 35; acute (single bout); healthy adults; laboratory |
| **Colón-Semenza et al. 2021** | Cross-sectional case–control; 32 Parkinson's patients vs 23 controls | Effort-based decision-making for exercise (cycling physical-effort task) | Cycling-based physical-effort task | No group differences: hard-choice proportion cycling PD 65.97% vs HC 64.37% ($p = .76$); button-press PD 59.08% vs HC 60.60% ($p = .78$); group status ns (cycling $\beta = 0.15$, $p = .74$; button $\beta = -0.62$, $p = .33$); cycling–button decisions correlated (Spearman $\rho = 0.562$, $p < .001$); motivation predicted decisions in PD | Small clinical sample; cross-sectional; mild–moderate/active PD limits generalisability |
| **Bieleke et al. 2025** | Scale development & validation; 3 studies, total N = 1267 (incl. longitudinal) | Value of Physical Effort (VoPE) self-report scale | Self-reported sport/exercise behaviour & physical activity | Reliability $\Omega = 0.89$ (95% CI 0.88–0.90); unidimensional ($\chi^2(2) = 0.54$, $p = .76$; RMSEA = .00); test–retest ICC = 0.77 (95% CI .71–.82); VoPE predicted sport/exercise behaviour (standardised βs significant, $p < .001$; $R^2$ up to ≈ .19), incl. longitudinal forecast | Self-report exposure & outcome (shared-method variance); largely cross-sectional |
| **Taylor et al. 2026** | Three-study forced-choice experiment; 2784 choices (Studies 1–2 hypothetical, Study 3 consequential) | Effort-based forced choices between exercise scenarios (effort duration/intensity) | Hypothetical (Tier 2) and performed (Tier 1) exercise choices | Greater effort reduced the exercise-option likelihood, moderated by autonomous motivation: effort duration low-autonomous $\beta = -.525$ (95% CI −.649 to −.401, $p < .001$), moderately autonomous $\beta = -.218$ (95% CI −.306 to −.130, $p < .001$), highly autonomous ns ($\beta = .09$, $p = .16$); effort | Mostly hypothetical choices; single sitting; generalisability of scenarios |

| | | | | | |
|---|---|---|---|---|---|
| | | | | intensity β = .535 (95% CI .411–.659, $p < .001$) | |
| **Cheval, Maltagliati et al. 2024** | Scale development & validation; 3 studies (Study 3 n = 297) | Physical Effort Scale (PES): approach/avoidance of physical effort | Self-reported usual physical-activity level | Subscale reliability α = .897 (approach) / .913 (avoidance); CFA fit CFI = .990; PES explained usual physical activity (approach b = 2.70, 95% CI 2.57–2.82; avoidance b = 3.24, 95% CI 3.10–3.38; adjusted $R^2 \approx .47$) | Self-report exposure & outcome (shared-method variance); cross-sectional |

Values are reported as given in each source. B/b = unstandardised coefficient; β = standardised coefficient; CI = confidence interval; d = Cohen's d; $\eta p^2$ = partial eta-squared; ICC = intraclass correlation; Ω = McDonald's omega; MVPA = moderate-to-vigorous physical activity; EEfRT = Effort-Expenditure for Rewards Task; SVC = small-volume corrected. Where a study reported only model-level or mediation significance, this is indicated. Full extraction (country, sex, age, follow-up, measurement details) is available on request.
